# The Colonization History of *Juniperus brevifolia* (Cupressaceae) in the Azores Islands

**DOI:** 10.1371/journal.pone.0027697

**Published:** 2011-11-16

**Authors:** Beatriz Rumeu, Juli Caujapé-Castells, José Luis Blanco-Pastor, Ruth Jaén-Molina, Manuel Nogales, Rui B. Elias, Pablo Vargas

**Affiliations:** 1 Island Ecology and Evolution Research Group, IPNA-CSIC, La Laguna, Tenerife, Canary Islands, Spain; 2 Department of Molecular Biodiversity and DNA Bank, Jardín Botánico Canario ‘Viera y Clavijo’ - Unidad Asociada CSIC, Tafira, Las Palmas de Gran Canaria, Spain; 3 Grupo da Biodiversidade dos Açores (CITA-A), Departamento de Ciências Agrárias, Universidade dos Açores, Angra do Heroísmo, Azores, Portugal; 4 Real Jardín Botánico, CSIC, Madrid, Spain; University of Lausanne, Switzerland

## Abstract

**Background:**

A central aim of island biogeography is to understand the colonization history of insular species using current distributions, fossil records and genetic diversity. Here, we analyze five plastid DNA regions of the endangered *Juniperus brevifolia*, which is endemic to the Azores archipelago.

**Methodology/Principal Findings:**

The phylogeny of the section *Juniperus* and the phylogeographic analyses of *J. brevifolia* based on the coalescence theory of allele (plastid) diversity suggest that: (1) a single introduction event likely occurred from Europe; (2) genetic diversification and inter-island dispersal postdated the emergence of the oldest island (Santa Maria, 8.12 Ma); (3) the genetic differentiation found in populations on the islands with higher age and smaller distance to the continent is significantly higher than that on the younger, more remote ones; (4) the high number of haplotypes observed (16), and the widespread distribution of the most frequent and ancestral ones across the archipelago, are indicating early diversification, demographic expansion, and recurrent dispersal. In contrast, restriction of six of the seven derived haplotypes to single islands is construed as reflecting significant isolation time prior to colonization.

**Conclusions/Significance:**

Our phylogeographic reconstruction points to the sequence of island emergence as the key factor to explain the distribution of plastid DNA variation. The reproductive traits of this juniper species (anemophily, ornithochory, multi-seeded cones), together with its broad ecological range, appear to be largely responsible for recurrent inter-island colonization of ancestral haplotypes. In contrast, certain delay in colonization of new haplotypes may reflect intraspecific habitat competition on islands where this juniper was already present.

## Introduction

Volcanic islands are geographic units that emerged from the ocean floor. After colonization by plants, oceanic barriers isolate island populations from the mainland and from each other, thus reducing gene flow [1], [2]. It is expected that plants displaying traits favorable for dispersal, establishment and distribution, such as anemophily, zoochory and self-compatibility, have been more successful in the colonization of remote archipelagos and their islands [3], [4].

**Figure 1:**
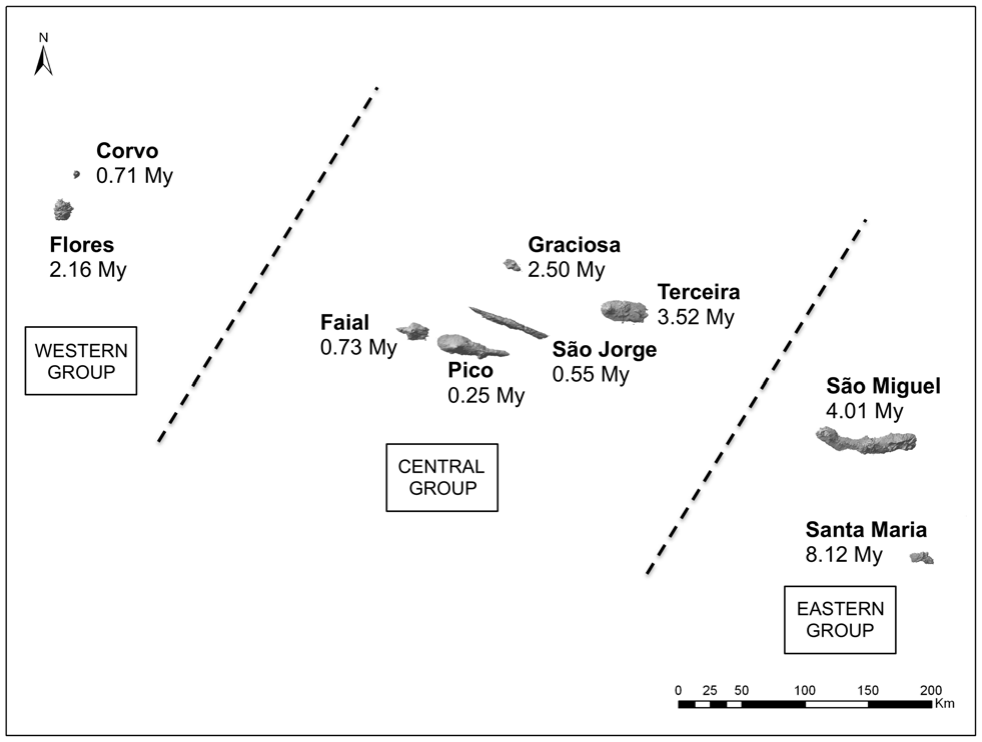
The Azores archipelago. Island groups and maximum geological ages according to França et al. [67].

Located in the Atlantic Ocean, the Macaronesian oceanic insular hotspot harbors three endemic juniper species (Cupressaceae), each distributed over a different archipelago: (1) *Juniperus brevifolia* (Seub.) Antoine in the Azores, (2) *J. cedrus* Webb & Berth. in the Canary Islands, and (3) *J. maderensis* (Menezes) R. P. Adams in Madeira [5]. These three taxa belong to the section *Juniperus*, which contains 11 of the ca. 67 species included in the genus [6]. Besides the Macaronesian junipers, the section comprises seven species currently distributed in the Mediterranean region and eastern Asia, plus *J. communis*, which has a widely circumboreal distribution. Their presence in the Atlantic archipelagos *per se* entails at least one long-distance dispersal event from the continent, though preliminary molecular phylogenies suggest at least two events (see [3]). In particular, the presence of *J. brevifolia* in the Azores implies one of the most remote juniper colonizations known to date (distance of about 1300 Km from western Europe, 1600 Km from eastern North America, and 800 km from north-west Madeira, which is the nearest Macaronesian archipelago). The Azores archipelago is located between 36°–40° N and 24°–32°W, and it comprises nine main islands of different geological ages, divided into Western (Corvo and Flores), Central (Faial, Pico, São Jorge, Graciosa and Terceira), and Eastern (São Miguel and Santa Maria) groups, with respective ages of 0.71–2.16 Mya, 0.25–3.52 Mya, and 4.01–8.12 Mya (Fig. 1). These islands are the result of the active volcanism associated with the divergence of the African, Eurasian and American tectonic plates. Lying over a 615 km long axis, the minimum distance between western and central groups is currently of 218 km, and of 139 km between central and eastern groups.

Fossil-calibrated phylogenetic studies suggest a high number of relict Tertiary lineages in Macaronesia [3], [7], [8]. However, molecular studies have also shown that several presumed Macaronesian relict lineages have a recent origin [1], [3]. As a general pattern, the presence of plants in oceanic islands probably is the result of many different colonization processes since the islands emerged, as revealed by the contrasting evolutionary histories of the lineages assessed. In particular, the colonization history of the remote archipelago of Azores is poorly understood. Even though some of the 149 native angiosperms [9] have been analyzed under phylogenetic perspectives (e.g. *Bellis* [10], *Euphorbia* [11], *Vaccinium* [12], *Pericallis* [13], *Lotus* [14], *Azorina* [15], *Euphrasia* [16], *Laurus* [17]), only a few of them have been the focus of deeper phylogeographic studies in the Azores archipelago (e.g. *Festuca* [18], *Picconia* [19], *Ammi*, *Angelica*, *Azorina*, *Euphorbia*, *Pericallis* [20]), far fewer phylogeographic results related to interisland colonization are known.

The study of representatives from different Azorean plant groups allows us to single out the most successful combinations of traits to thrive in these remote islands, and provide a general framework to understand the different processes that foster colonization. Regardless of being dioecious and, hence, self-incompatible, *J. brevifolia* represents one of the best examples of endemic species displaying traits favorable for long-distance dispersal and colonization, i. e. anemophily, zoochory, multi-seeded cones [6] and mesic habitat requirements [21], among others. If *J. brevifolia* derives from a recent colonization, low levels of genetic diversity would be expected overall, whereas if colonization by *J. brevifolia*'s ancestor occurred far back in time we would expect that extant populations have high genetic diversity between island populations due to genetic drift favoring/fixing different alleles and haplotypes [22], [23]. Consequently, DNA data may provide us with important variables to reconstruct the chronological sequence of diversification in the Azorean juniper.

In angiosperms, both plastid DNA (cpDNA) and mitochondrial DNA (mtDNA) are maternally inherited in most cases [24], and the degree of genetic structuring shaped by organelle DNA can only be interpreted in terms of seed dispersal. However, previous conifer studies show predominant paternal inheritance of cpDNA in this group and in members of the Cupressaceae family [24]–[28]. Furthermore, because the spatial pattern of the adult plants is a consequence of the seed dispersal [29], molecular variables make it possible to relate the haplotype distribution of the Azores juniper to the contribution of both seed and pollen gene flow.

In this investigation, we used plastid DNA sequences of *J. brevifolia* to: (1) infer the temporal and spatial origin, (2) estimate genetic diversity levels on each island, and (3) reconstruct the phylogeographic history in the Azores archipelago.

## Methods

### Study species

As the remaining juniper species, *J. brevifolia* is dioecious and wind-pollinated, shedding pollen from the male cones principally during spring [6]. It develops fleshy female cones that ripen in summer and autumn [30], and are consumed mainly by birds [31], as recorded for continental congeners [32]–[35].

As a consequence of deforestation since the onset of human settlement in the 15th century, natural populations of the two Azorean native conifers (*Taxus baccata* and *J. brevifolia*) have been drastically reduced. Thus, Schirone et al. [36] predict imminent extinction for *T. baccata* and report only 5 living individuals on Pico island, whereas *J. brevifolia* populations are nowadays scarce and/or fragmented and the species is considered as ‘endangered’ on a global scale [37]. Notably, although *J. brevifolia*'s range is much more restricted than in the past, and it is nowadays extinct on Graciosa and critically endangered on Santa Maria, its current distribution entails a wide ecological range, and there are still extensive natural areas on some islands where plant communities are dominated by this juniper [21], [38].

### Ethics statement

The ‘Secretaria Regional do Ambiente e do Mar - Direcção Regional do Ambiente’ from the Azorean Autonomous Region provided us with the required permit for the collection of wild plant leaves (LICENÇA N° 59/2008/DRA).

### Plant material and DNA plastid sequencing

For the inference of intraspecific patterns of *J. brevifolia* cpDNA sequence variation, needles were collected from trees on all the islands of occurrence. Except for Santa Maria, where only two samples could be collected, about 50 trees were sampled from each island. The geographic coordinates of each sample were recorded using a hand-held GPS navigator. In total, needles of 367 trees were sampled and stored in zippered plastic bags containing silica gel. Total DNAs were extracted from silica-gel dried needles using the CTAB 2x method [39], [40]. The concentration of the total DNA obtained was measured in an Eppendorf biophotometer, and its quality assessed in 1% agarose gels.

To perform phylogenetic analyses of section *Juniperus*, we took *trn*L intron and *trn*L-*trn*F intergenic spacer [41] sequences from previous studies ([42], Martínez and Vargas, in prep.). A matrix using these cpDNA regions was constructed with nine *trn*L and *trn*L*-trn*F sequences from Mao et al. [42], and 36 *trn*L-*trn*F sequences from Martínez and Vargas (unpublished data); the latter were completed with their corresponding 36 *trn*L sequences, which were newly generated by us for this study. In addition, the matrix was extended with two samples of the Macaronesian junipers *J. cedrus* and *J. maderensis* and eight samples of our focal species (*J. brevifolia*). Based on previous phylogenetic analyses of *Juniperus* [42], we also added sequences of three outgroup samples of *J. drupacea* (section *Caryocedrus*) (Tables 1/S1). PCR conditions were based on Taberlet et al. [41] with some variations: 95°C for 1 min, followed by 35 cycles of 94°C for 30 s, 56°C for 2 min, and 72°C for 2 min, with a final extension step of 72°C for 10 min.

**Table 1:** Taxon names and origin of the *Juniperus* samples included in the phylogenetic analysis.

Coding no.	Taxon	Origin
1	*J. brevifolia*	Portugal, Azores, Santa Maria, Almagreira
2	*J. brevifolia*	Portugal, Azores, São Miguel, Serra da Tronqueira
3	*J. brevifolia*	Portugal, Azores, Terceira, Malha Grande
4	*J. brevifolia*	Portugal, Azores, São Jorge, R.F. Pico da Esperança
5	*J. brevifolia*	Portugal, Azores, Pico, Cachorro, BR4347
6	*J. brevifolia*	Portugal, Azores, Pico, Lagoa do Capitão
7	*J. brevifolia*	Portugal, Azores, Faial, Caldeira
8	*J. brevifolia*	Portugal, Azores, Faial, Caldeira
9	*J. brevifolia*	Portugal, Azores, Flores, Alto da Cova
10	*J. brevifolia*	Portugal, Azores, Corvo, Lomba Redonda
11	*J. cedrus*	Spain, Canary Islands, Tenerife, La Orotava
12	*J. cedrus*	Spain, Canary Islands, La Palma, Pared de Roberto
13	*J. communis* var. *communis*	Spain, Burgos, Covarrubias
14	*J. communis* var. *communis*	Spain, Granada, Pico Trevenque
15	*J. communis* var. *communis*	France
16	*J. communis* var. *depressa*	USA, New Mexico
17	*J. communis* var. *saxatilis*	Spain, Madrid, La Pedriza
18	*J. communis* var. *saxatilis*	Georgia, Caucasus
19	*J. communis* var. *saxatilis*	Pakistan
20	*J. deltoides*	Turkey
21	*J. formosana* var. *mairei*	China, Gansu
22	*J. macrocarpa*	Spain, Cádiz, Barbate, Trafalgar Cape
23	*J. macrocarpa*	Spain, Cádiz, Tarifa
24	*J. macrocarpa*	Spain, Valencia, El Saler
25	*J. macrocarpa*	Spain, Cádiz, Chiclana
26	*J. macrocarpa*	Italy, Sardinia, Santa Teresa Gallura
27	*J. macrocarpa*	Italy, Sicily, Ragusa
28	*J. maderensis*	Madeira, Fajã da Nogueira
29	*J. navicularis*	Portugal, Apostiça
30	*J. navicularis*	Portugal, Apostiça
31	*J. navicularis*	Portugal, Estremadura
32	*J. oxycedrus* var. *badia*	Spain, Huesca, Sierra de Guara
33	*J. oxycedrus* var. *badia*	Spain, Madrid, Villalba
34	*J. oxycedrus* var. *badia*	Spain, Ciudad Real, Puebla de Don Rodrigo
35	*J. oxycedrus* var. *badia*	Spain, Jaén
36	*J. oxycedrus* var. *badia*	Spain, Jaén
37	*J. oxycedrus* var. *badia*	Morocco, Kjbel Kelti
38	*J. oxycedrus* var. *badia*	Morocco, Kjbel Kelti
39	*J. oxycedrus* var. *badia*	Turkey, Gümüshane, Torul
40	*J. oxycedrus* var. *oxycedrus*	Spain, Granada, El Peñón
41	*J. oxycedrus* var. *oxycedrus*	Spain, Granada, El Peñón
42	*J. oxycedrus* var. *oxycedrus*	Spain, Balearic Islands, Menorca
43	*J. oxycedrus* var. *oxycedrus*	Greece, Lemo
44	*J. oxycedrus* var. *oxycedrus*	Greece, Lemo
45	*J. oxycedrus*	Turkey, Istanbul, Kartal
46	*J. oxycedrus*	Greece, Kalavryta, Diakoftó
47	*J. oxycedrus*	Tunisia, Cap Bon, Sidi Daoud
48	*J. oxycedrus*	France
49	*J. rigida* var. *conferta*	Cultivated (Spain, Pontevedra, Lourizán)
50	*J. rigida* var. *conferta*	Japan
51	*J. rigida* var. *rigida*	Japan
52	*J. rigida* var. *rigida*	Japan
53	*J. taxifolia*	Japan
54	*J. taxifolia*	Japan, Bonin Island
55	*J. taxifolia* var. *lutchuensis*	Japan
	OUTGROUP	
56	*J. drupacea*	Greece
57	*J. drupacea*	Greece
58	*J. drupacea*	Greece

**Figure 2:**
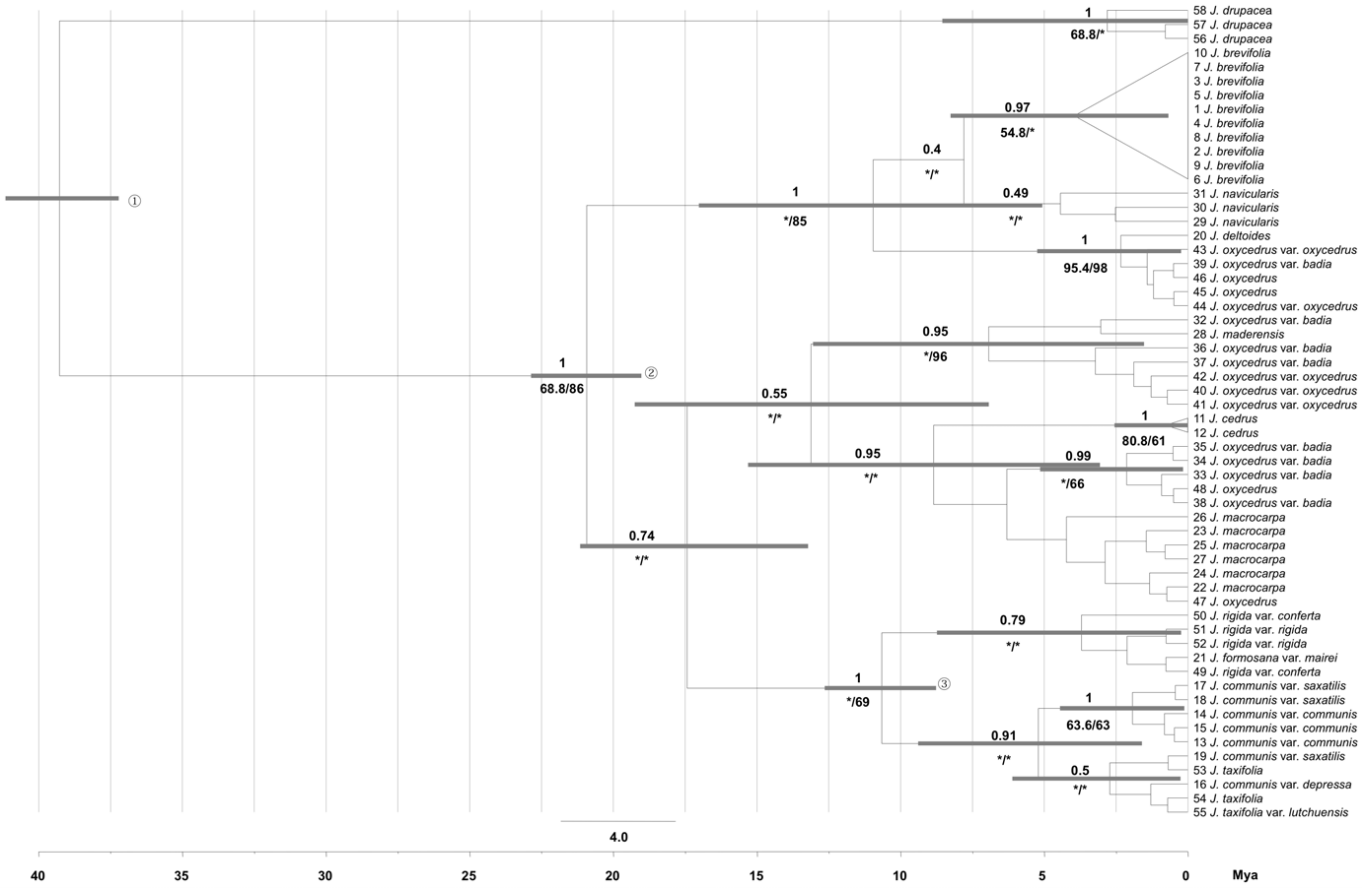
Phylogenetic relationships within *Juniperus* section inferred from *trn*L and *trn*L-*trn*F, and divergence time-scale derived from BEAST. Numbers before taxon names refer to the coding no. given in Tables 1/S1. Numbers above branches are BEAST posterior probabilities; numbers below branches are Maximum Likelihood bootstrap support values (before slashes); and Maximum Parsimony bootstrap support values (after slashes). Asterisks indicate absence of support. Gray bars represent divergence times (95% highest posterior density intervals) for each node, while numbers in white circles represent calibration points obtained from Mao et al. [42]: (1) split between sects. *Juniperus*-*Caryocedrus* (49.1–29.9 Mya), (2) crown of sect. *Juniperus* (29.9–11.1 Mya) and (3) crown of BSG in sect. *Juniperus* (17.5–4.7 Mya). BEAST posterior probability values for calibration nodes were inferred from Mao et al. [42].

Coding numbers as in Fig. 2. Taxonomy follows that of [6]. See Table S1 for details.

To perform phylogeographic analyses of *J. brevifolia*, we tested two to eight individuals from different islands for 19 plastid DNA regions in an exploratory study based on previous phylogenetic and phylogeographic analyses [41], [43]–[51]. The three most variable plastid DNA regions (*pet*N-*psb*M [43], *trn*S-*trn*G [45], *trn*T-*trn*L [41]) were used to sequence 9–10 individuals per island (except for Santa Maria, where only two trees are known) in order to assemble the phylogeographic matrix (Tables 2/S2). PCR protocols varied slightly for the three DNA regions, and consisted of: 30–35 cycles of 94°C for 30 s, 50–56°C for 2 min and 72°C for 2 min, preceded by an initial denaturation at 95°C for 1 min and followed by a final extension at 72°C for 5–10 min. A volume of 1 µl of bovine serum albumin (BSA) at 1 mg ml^−1^ was included in each 25 µl reaction to improve the efficiency of the amplification. PCR products were sequenced using an ABI Prism ® 3730xi DNA sequencer at the Macrogen Institute (Macrogen Co., Korea). Sequences were aligned and manually adjusted using MAFFT v6.814b implemented in the Geneious 5.1.7 software. All new sequences have been deposited in GenBank (see Tables S1 and S2 for accession numbers).

**Table 2:** Plant material of *Juniperus brevifolia* used for sequencing *pet*N-*psb*M, *trn*S-*trn*G and *trn*T-*trn*L from 71 samples.

Geographical area/locality	N	H
Portugal, Azores, Santa Maria, Almagreira	2	9
Portugal, Azores, São Miguel, Monte Escuro	4	8, 11, 9
Portugal, Azores, São Miguel, Serra da Tronqueira	6	12, 9, 14, 10
Portugal, Azores, Terceira, Malha Grande	3	8, 16, 13
Portugal, Azores, Terceira, Pico Alto	3	8, 16, 7
Portugal, Azores, Terceira, Santa Bárbara	2	17, 9
Portugal, Azores, Terceira, Fajãzinha	2	16
Portugal, Azores, São Jorge, Bocas do Fogo	2	8, 9
Portugal, Azores, São Jorge, R.F. Pico da Esperança	4	14, 8, 4
Portugal, Azores, São Jorge, Serra do Topo	4	8, 15
Portugal, Azores, Pico, Cachorro	1	8
Portugal, Azores, Pico, Cerrado de Sonicas	1	14
Portugal, Azores, Pico, Montanha	1	4
Portugal, Azores, Pico, Baldios	1	4
Portugal, Azores, Pico, Lagoa do Capitão	1	8
Portugal, Azores, Pico, Curral Queimado-R.F. Prainha W	1	8
Portugal, Azores, Pico, Curral Queimado-R.F. Prainha	1	6
Portugal, Azores, Pico, Piquete do Caveiro W	1	4
Portugal, Azores, Pico, Manhenha	1	8
Portugal, Azores, Faial, Quebrada	2	8
Portugal, Azores, Faial, Grotão	1	8
Portugal, Azores, Faial, Caldeira	4	8, 6, 3
Portugal, Azores, Faial, Cabeço dos Trinta	3	4, 8, 5
Portugal, Azores, Flores, Lagoa	1	8
Portugal, Azores, Flores, Alto da Cova	2	8
Portugal, Azores, Flores, Pico da Casinha	1	8
Portugal, Azores, Flores, Caldeirães	1	8
Portugal, Azores, Flores, Morro Alto e Pico da Sé	2	5, 14
Portugal, Azores, Flores, Caldeira Funda e Rasa	2	2, 8
Portugal, Azores, Flores, Fajãzinha	1	8
Portugal, Azores, Corvo, Lomba Redonda	6	14, 8, 6
Portugal, Azores, Corvo, Cabeçeira	2	14, 16
Portugal, Azores, Corvo, Alqueve	2	14
OUTGROUP		
Greece, Lemo	1	1

**Figure 3:**
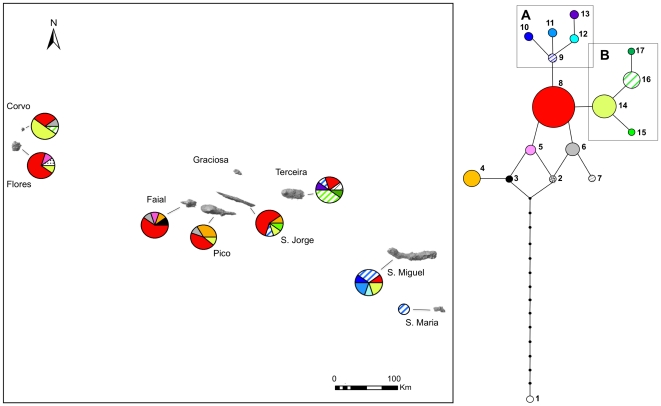
cpDNA (*pet*N-*psb*M/*trn*S-*trn*G/*trn*T-*trn*L) haplotype network and its spatial distribution in the Azores archipelago. Each haplotype is represented by both a number and a color. Haplotype sizes are proportional to the number of individuals displaying them. Distinct clades (A and B) are shown within boxes.

N is the number of samples collected in each locality and H corresponds to the haplotypes found according to Fig. 3. Outgroup taxon (last row): *Juniperus oxycedrus* var. *oxycedrus*. See Table S2 for voucher source, haplotype number of each tree sampled, and the GenBank accession numbers of each haplotype found.

To extend the phylogeographic analysis, we also amplified the *psb*A-*trn*H region [48] (following the above-mentioned conditions, but without using BSA). In this case, the presence of a 58 bp insertion/deletion detected in the preliminary screening was checked in 1.5% agarose gels for the 367 samples collected in the field (Table 3).

**Table 3:** *Juniperus brevifolia* individuals (n = 367) used to infer the amplicon length of the *psb*A-*trn*H region.

Geographical area/Locality	N	Vouchers	psbA-trnH amplicon length (bp)
Azores, Santa Maria, Almagreira	2	BR4710, BR4711	2 (466)
Azores, São Miguel, Monte Escuro	15	BR4494–BR4508	15 (466)
Azores, São Miguel, Serra da Tronqueira	35	BR4509–BR4543	1 (408); 34 (466)
Azores, Terceira, Malha Grande	20	BR4544–BR4563	5 (408); 15 (466)
Azores, Terceira, Pico Alto	20	BR4564–BR4583	3 (408); 17 (466)
Azores, Terceira, Santa Barbara	20	BR4584–BR4603	6 (408); 14 (466)
Azores, Terceira, Fajãzinha	6	BR4604–BR4608	1 (408); 5 (466)
Azores, São Jorge, Bocas do Fogo	2	BR4444, BR4445	2 (466)
Azores, São Jorge, R. F. Pico da Esperança	21	BR4446–BR4466	3 (408); 18 (466)
Azores, São Jorge, Serra do Topo	27	BR4467–BR4493	4 (408); 23 (466)
Azores, Pico, Cachorro	4	BR4344–BR4347	2 (408); 2 (466)
Azores, Pico, Cerrado dos Sonicas	8	BR4348–BR4353; BR4359–BR4360	4 (408); 4 (466)
Azores, Pico, Montanha	5	BR4354–BR4358	4 (408); 1 (466)
Azores, Pico, Baldios	2	BR4361–BR4362	1 (408); 1 (466)
Azores, Pico, Lagoa do Capitão	9	BR4363–BR4371	2 (408); 7 (466)
Azores, Pico, Curral Queimado–R. F. Prainha W	2	BR4372–BR4373	2 (408)
Azores, Pico, Curral Queimado-R. F. Prainha	5	BR4374–BR4378	3 (408); 2 (466)
Azores, Pico, Curral Queimado-R. F. Prainha E	2	BR4379–BR4380	2 (466)
Azores, Pico, Piquete do Caveiro W	6	BR4381–BR4386	1 (408); 5 (466)
Azores, Pico, Piquete do Caveiro E	2	BR4387–BR4388	2 (466)
Azores, Pico, Piquete do Caveiro S	1	BR4389	1 (466)
Azores, Pico, Manhenha	4	BR4390–BR4393	4 (466)
Azores, Faial, Quebrada	10	BR4395–BR4402	1 (408); 9 (466)
Azores, Faial, Grotão	4	BR4404–BR4406; BR4424	4 (466)
Azores, Faial, Caldeira	17	BR4497–BR4423	3 (408); 14 (466)
Azores, Faial, Cabeço dos Trinta	19	BR4425–BR4443	4 (408); 15 (466)
Azores, Flores, Lagoa	1	BR4660	1 (466)
Azores, Flores, Alto da Cova	18	BR4661–BR4678	1 (408); 17 (466)
Azores, Flores, Pico da Casinha	5	BR4679–BR4683	5 (466)
Azores, Flores, Caldeirães	2	BR4684–BR4685	2 (466)
Azores, Flores, Morro Alto e Pico da Sé	7	BR4686–BR4692	7 (466)
Azores, Flores, Caldeira Funda e Rasa	8	BR4693–BR4700	8 (466)
Azores, Flores, Fajãzinha	9	BR4701–BR4709	9 (466)
Azores, Corvo, Lomba Redonda	27	BR4610–BR4637	27 (466)
Azores, Corvo, Cabeceira	17	BR4638–4654	17 (466)
Azores, Corvo, Alqueve	5	BR4655–4659	5 (466)

N: number of individuals sampled in each locality. Voucher abbreviations: BR: B. Rumeu collection numbers as coded in the DNA Bank at the Jardín Botánico Canario ‘Viera y Clavijo’- Unidad Asociada-CSIC. Length of the *psb*A-*trn*H amplicon is shown in the last column: number of individuals without brackets, amplicon length (bp) in brackets. Representative sequences of the two amplicon types of different lengths were deposited in the GenBank (JF951047, 408 bp; JF951048, 466 bp).

### Data analysis

To obtain the taxonomic relationships within *Juniperus* section *Juniperus* and to infer the temporal and spatial origin of *J. brevifolia*, Maximum Likelihood (ML), Maximum Parsimony (MP) and Bayesian Inference (BI) analyses were conducted for the *trnL* intron and *trnL-trn*F dataset. Large indels were found in the *trn*L-*trn*F spacer; as gap characters represent in this case a considerable portion of the potential phylogenetic information [52], all indels detected were manually coded following the method of Simmons and Ochoterena [52] and included in the analyses. To determine the model of sequence evolution that best fits the sequence data (F81+G), the Akaike Information Criterion (AIC; [53]) was implemented in this dataset using jModeltest 0.1.1 [54]. ML analyses were performed using PhyML [55] including the model parameters previously obtained with jModeltest. ML bootstrap analysis was carried out with the same software and settings, using 500 non-parametric bootstrap replicates. Parsimony analyses were run in TNT 1.1 [56] under traditional heuristic search. We first used the Tree Bisection-Reconnection (TBR) branch-swapping algorithm with 10,000 replicates (saving two most-parsimonious trees per replicate); subsequently, the trees obtained in the first search were used to start a second heuristic search that retained all best trees. Branch support was evaluated using 1,000 bootstrap replicates, collapsing groups with branch support values below 50. The BI analysis was used to estimate divergence times within section *Juniperus,* and of *J. brevifolia*. We implemented a relaxed molecular-clock approach in BEAST v.1.6.0 [57], [58], which simultaneously estimates phylogenetic relationships and node ages. The molecular clock analysis was carried out with two data partitions: (1) the *trn*L intron and *trn*L-*trn*F intergenic spacer dataset (as only two nucleotide substitution models were available in BEAST v.1.6.0, we used the HKY+G model as the closest to our dataset following the AIC criterion), and (2) indels from partition 1 coded as binary data (binary simple substitution model [52]) using the software SeqState 1.4.1 [59]. For the temporal calibration we used several divergence times formerly obtained by Mao et al. [42]: (1) the split between sections *Juniperus*-*Caryocedrus* (49.1–29.9 Mya), (2) the crown of section *Juniperus* (29.9–11.1 Mya), and (3) the crown of the ‘blue seed cone group’ (BSG) in section *Juniperus* (17.5–4.7 Mya). The substitution rate variation was modeled using an uncorrelated lognormal distribution, and a Birth-Death process [60] was employed as tree prior. Two MCMC analyses were run for 10 million generations with a sample frequency of 1,000, and discarding the first 10% generations as burn-in. Analysis with Tracer 1.4 [61] confirmed adequate sample size, with ESS values above 200. Both analyses were combined using LogCombiner 1.4.8, and trees were summarized in a maximum clade credibility tree obtained in TreeAnotator 1.4.8.

To infer connectivity between island populations of *J. brevifolia*, the *pet*N-*psb*M, *trn*S-*trn*G and *trn*T-*trnL* sequences obtained were concatenated, and a single analysis was performed based on the common inheritance without recombination that can be assumed for cpDNA markers [62]. One of the species (*J. oxycedrus*) most closely related to *J. brevifolia* according to the phylogenetic analysis was also included. Here, indels were also manually coded following Simmons and Ochoterena [52]. We performed a phylogeographic analysis based on the coalescence theory [63]. A statistical parsimony method [64] implemented in the TCS 1.21 software [65] was used to infer genealogical relationships among haplotypes. The maximum number of differences resulting from single substitutions among haplotypes was calculated with 95% confidence limits, treating gaps as the fifth state.

The nearest-neighbor statistic (Snn) was calculated to assess genetic differentiation in *J. brevifolia*, as we expected isolation by distance among island groups, and also due to temporal differences in island emergence. This statistic is a measure of how often the ‘nearest neighbors’ (similar sequences) belong to the same pre-defined cluster [66]. The closer Snn is to 1, the more differentiated are the populations within the partitions of a dataset; if Snn is close to 0.5, the partitions are construed as components of a single panmictic population. To detect the genetic differentiation attributable to geography, the combined *pet*N-*psb*M, *trn*S-*trn*G and *trn*T-*trn*L dataset of *J. brevifolia* was split into three according to the geographic island groups (Fig. 1). To assess the chronological component of genetic differentiation, the dataset was partitioned into two age groups: islands emerging before the Pleistocene (Santa Maria, São Miguel and Terceira; >2.5 Mya), and during the Pleistocene (Pico, Faial, São Jorge, Flores and Corvo; <2.5 Mya) [67]. In both cases, Snn was calculated using DnaSP v5 [68], with indels previously coded. Permutation tests with 1,000 replicates were performed to evaluate significance, and the Bonferroni correction for multiple comparisons to control for the occurrence of Type I-error.

In order to determine the statistical relationship between genetic (F_ST_) and geographic distances (in km) between all possible population pairs, we ran a Mantel test [69] as implemented in GENALEX 6.3 [70]. We considered islands as single populations, and measured the distances between them using straight-line distances in ArcGis 9.3 (Environmental Systems Research Institute, Redlands, CA). F_STs_ were calculated using DnaSP v5 [68].

To assess the geospatial distribution and diffusion process of *J. brevifolia* through time, an additional BEAST analysis was performed with the *pet*N-*psb*M, *trn*S-*trn*G, *trn*T-*trn*L dataset. The molecular clock analysis was also carried in two data partitions: (1) the concatenated *pet*N-*psb*M, *trn*S-*trn*G, *trn*T-*trn*L dataset (HKY+G nucleotide substitution model), and (2) indels from the previous partition (1) coded as binary data (binary simple substitution model [52]) using the the software SeqState 1.4.1 [59]. For the temporal calibration, we set the diversification time of *J. brevifolia* as 8.27–0.69 Mya, obtained in the previous molecular dating analysis. Hence, we constrained the crown node of *J. brevifolia* to this age (mean 4.48, SD 2.305 with normal distribution). The substitution rate variation was modeled with an uncorrelated lognormal distribution, and a coalescent (constant size) tree prior was employed. Four MCMC analyses were run for 10 million generations, with a sample frequency of 1,000, discarding the first 10% generations as burn-in. We also confirmed adequate sample size after combining the four analyses with LogCombiner 1.4.8. Following the instructions described in http://beast.bio.ed.ac.uk, the BEAUti file (.xml) was modified assigning a fixed spatial location to each sample (here, eight locations for the ingroup, corresponding to each of the Azorean islands sampled). Finally, a discrete phylogeographic analysis was performed using a standard continuous-time Markov chain as described in Lemey et al. [71]. This analysis determined the probability distribution of the eight locations in the nodes of the Maximum clade credibility tree. A Bayesian stochastic variable selection model (BSSVS, which is an extension of the discrete phylogeographic model) using the Bayes Factor (BF) test allowed us to achieve statistical significance for the rates of the dispersal events. Using a tool added to the BEAST code (RateIndicatorBF), we visualized the well-supported rates of dispersal in Google Earth.

## Results

### Phylogenetic analyses and lineage divergence times

Phylogenetic relationships using *Juniperus* sequences of the *trn*L intron and the *trn*L*-trn*F region and Bayesian (BEAST) and ML analyses suggested the monophyly of *J. brevifolia*, albeit this result was not supported by the MP analysis (Fig. 2). The diversification time of *J. brevifolia* was estimated to occur at between 8.27–0.69 Mya. In addition, all three analyses pointed to a sister group of closely related species from Portugal (*J. navicularis*) and the eastern Mediterranean basin (*J. deltoides* and populations of *J. oxycedrus*). The divergence time between the clade of *J. brevifolia* + *J. navicularis* and its sister group was estimated at between 17.03–5.07 Mya (95% highest posterior density interval), in the upper Miocene – lower Pliocene.

### Haplotype networking

Within *J. brevifolia*, we detected 16 haplotypes as a result of variation of *pet*N-*psb*M, *trn*S-*trn*G and *trn*T-*trn*L sequences in four nucleotide substitutions and seven gaps. The resulting indels (treated as a fifth character) were distributed as follows: in *pet*N-*psb*M, 14 bp between 216–229, 1 bp at position 563, 24 bp between positions 566–589; in *trn*S-*trn*G, 120 bp between 331–450; in *trn*T-*trn*L, 2 bp between 266–267, 1 bp at position 267 and 1 bp at 303. Table S2 shows the haplotype found in each locality; for the haplotype distribution of the 72 samples analyzed, see Table S2. The statistic parsimony analysis connected the outgroup accession of *J. oxycedrus* var. *oxycedrus* (haplotype 1) with the network of *J. brevifolia*, needing 14 mutation steps. Only two loops were retrieved, while two distinct clades (A and B) were obtained (Fig 3). The internal haplotypes 8 and 14 are widely distributed over the three island groups, whereas the other internal haplotypes are shared between the central and the western groups (5, 6, 16), and between the central and eastern groups (9). Six tip haplotypes are exclusive of a single island: Terceira (7, 13, 17), São Miguel (10, 11) and São Jorge (15). A lineage formed by haplotypes 9, 10, 11, 12 and 13 was clearly associated with the easternmost islands.

### *psb*A*-trn*H length polymorphism in *J. brevifolia*

Two different fragments of the *psb*A-*trn*H region were detected when analyzing the 367 *J. brevifolia* trees (Table 3), with lengths of 408 bp and 466 bp. None of the 99 trees from the western group, and only one of the 52 trees from the eastern group, showed the 408 bp amplicon. However, high levels of sequence length variation were detected in samples from the central group, where 28.6% of trees displayed the shorter fragment, and 71.4% the longer one. Across islands, the presence of the shorter amplicon in the samples from the central group was distributed as follows: 22.7% on Terceira, 14.0% São Jorge, 36% Pico and 16% Faial.

### Genetic differentiation analysis and Mantel Test

Values of Snn (Table 4) rendered significant results when comparing the genetic differentiation of the three island groups (eastern, central and western). We detected a highly significant genetic differentiation of eastern *J. brevifolia* populations with respect to the rest of the archipelago. However, no significant differences were found when comparing the two other island groups with the rest of the archipelago. There was also a highly significant genetic differentiation associated with the ages of the islands predating the Pleistocene vs. those that emerged during this period.

**Table 4:** Genetic differentiation associated with isolation according to island group distances and island ages.

	S_nn_
**Isolation by island group distances**	
Eastern group – Central group – Western group	0.52, p = 0.002**
Eastern group – Rest	0.85, p<0.001^(^***^)^
Central group – Rest	0.57, p = 0.032 (ns)
Western group – Rest	0.62, p = 0.109 (ns)
**Isolation by island ages**	
Predating Pleistocene – Postdating Pleistocene	0.79, p<0.001***

Nearest-neighbor statistic (Snn) values calculated from the combined *pet*N-*psb*M, *trn*S-*trn*G and *trn*T-*trn*L dataset of *J. brevifolia*. Significance levels in brackets were those obtained after Bonferroni correction.

ns, not significant; *, 0.01<P<0.05; **, 0.001<P<0.01; ***, P<0.001.

(Significant level after Bonferroni correction = 0.017)

The Mantel test between geographic distance and F_ST_ values revealed a weak but significant isolation-by-island distance effect, indicated by a low relationship between geographic distance and global F_ST_ values (1000 permutations, R^2^ = 0.38, p = 0.001) (Table 5).

**Table 5:** Population pairwise F_ST_ estimates based on haplotype sequences (above the diagonal) and geographic distances (in km, below the diagonal).

	MA	SM	TE	JO	PI	FA	FL	CO
MA	-	0.123	0.473	0.630	0.696	0.696	0.730	0.741
SM	92	-	0.200	0.188	0.304	0.309	0.297	0.325
TE	270	198	-	0.129	0.198	0.228	0.189	0.047
JO	306	245	60	-	-0.006	0.028	0.005	0.110
PI	325	271	95	36	-	-0.031	0.067	0.218
FA	362	308	127	68	38	-	-0.042	0.280
FL	599	543	350	298	274	236	-	0.203
CO	603	543	348	299	277	240	30	-

Islands have been considered as single *Juniperus brevifolia* populations and are abbreviated as follows: MA, Santa Maria; SM, São Miguel; TE, Terceira; JO, São Jorge; PI, Pico; FA, Faial; FL, Flores; CO, Corvo.

Negative values should be interpreted as no genetic differentiation between populations from the two islands and likely reflects the imprecision of the algorithm used by the software to estimate this value.

### Discrete Phylogeographic Analysis

**Figure 4:**
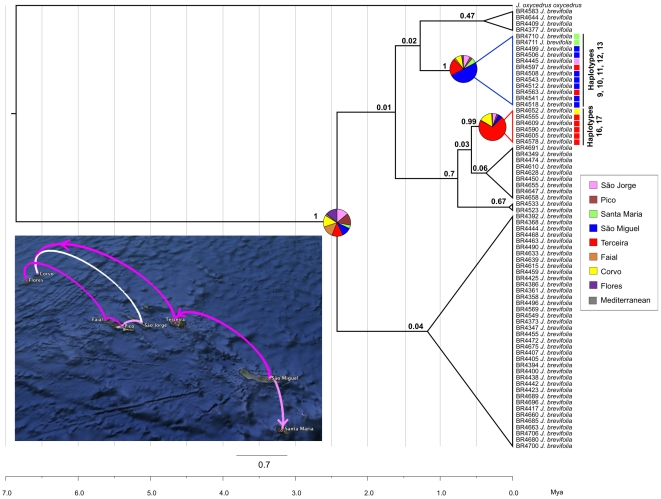
Relaxed molecular-clock chronogram and phylogeographic reconstruction of *Juniperus brevifolia.* Maximum clade credibility tree summarized from the geospatial Bayesian analysis of cpDNA (*pet*N-*psb*M, *trn*S-*trn*G and *trn*T-*trn*L sequences) of 71 individuals of *J. brevifolia*. Pie charts represent posterior probability distributions of ancestral range at well-supported nodes of interest. Colored rectangles represent the sample's island of origin. The haplotype relatedness is also shown in the well-supported clades. Colonization routes supported by a BF>3 are shown on the map. The color of each route represents its relative support, with more intense colors indicating stronger support. Arrows specify directionality in the colonization route, inferred from well-supported nodes of interest in the geospatial Bayesian analysis. The map is based on satellite images available in Google Earth (http://earth.google.com).

The Bayesian maximum clade credibility tree of the *pet*N-*psb*M, *trn*S-*trn*G and *trn*T-*trn*L dataset showed a considerably uncertainty in the geographic origin of *J. brevifolia* (Fig. 4). For the most ancestral supported node, a wide diversification time window of 4.99-0.65 Ma was estimated (data not shown). The only highly supported lineages indicated a most probable ancestral range in São Miguel for haplotypes 9, 10, 11, 12 and 13, and in Terceira for haplotypes 16 and 17. Six main migration routes were supported by the BF test (Fig 4): Terceira-Corvo (BF = 11.5), São Miguel-Terceira (BF = 11.34), Pico-Faial (BF = 9.75), Faial-Flores (BF = 9.62), Santa Maria- São Miguel (BF = 6.29), Pico-São Jorge (BF = 5.92) and São Jorge-Corvo (BF = 4.14). Both analyses support (1) the importance of Terceira as a stepping-stone island within the Azores, and (2) an east-west colonization of the haplotypes 9 and 16.

## Discussion

The results support a hypothesis in which a single introduction event likely from Europe, followed by inter-island dispersal, explains best the current distribution of *J. brevifolia*. However, DNA sequence data failed to support a clear sister-group relationship hypothesis, which prevented from estimating a particular time of colonization from the continent. This colonization may have been favored by the occurrence of several ancient islands (60–0.018 Mya), that could have acted as stepping-stones between continental Europe and the Azorean islands [72]. Nevertheless, divergence time estimates revealed that genetic differentiation of *J. brevifolia* postdated the emergence of the oldest island of Santa Maria (8.12 Mya [67]).

### High diversity and dynamic colonization on the oldest islands

Apart from Santa Maria, which has been dramatically deforested for centuries, and where only two trees could be found and sampled, a high diversity of haplotypes has been detected in the remaining seven islands. The easternmost Azorean islands harbor the highest diversity levels with six haplotypes (São Miguel, Terceira), followed by São Jorge and Faial with five, and the remaining three islands with four. These findings agree with Carine and Schaefer's [73] hypothesis of the ‘Azorean diversity enigma’, whereby most of the endemics are widespread across the archipelago. Moreover, they also agree with Schaefer et al. [20], who show that a range of Azorean endemic plant lineages contain high levels of intra-specific genetic variation comparable to (or even higher than) those found among the abundant congeneric single island endemics from the Canary Islands (e.g. Rumeu et al. for the Canarian juniper, unpublished).

The key role of São Miguel and Terceira in the diversification of *J. brevifolia* is also evidenced by the discrete phylogeographic analysis, which points to these two islands as the source of seven different haplotypes (Fig. 4). Notably, São Miguel and Terceira are the oldest islands (both predating the Pleistocene) with still large populations and also the closest to the continent, which suggests the hypothesis that either age or distance from the continent have been of paramount importance for early dispersal and establishment of *J. brevifolia* in the Azores. A similar positive correlation between genetic diversity and island age or proximity to the continent has been detected for the Canaries based on allozyme diversity, thus far the largest population genetics database for these islands' flora [74]. Results derived from our Snn analysis revealed significant genetic differentiation due to isolation-by-distance of the eastern group of the Azores with respect to the other two island groups, and also on a time-scale (i. e., considering whether islands emerged before or during the Pleistocene). However, temporal isolation rather than isolation-by-distance from the continent appears to have played a more determinant role for the first colonization, given similar geographic distances between island groups (Santa Maria - São Miguel, Terceira - São Jorge - Pico - Faial) and the mainland in a large scale.

Combining these results with those obtained by the BF test, it is possible to underscore the importance of Terceira in fostering genetic connectivity within the archipelago. Despite the migration route from Terceira to Corvo being well supported by the Discrete Phylogeographic Analysis and the BF test, it is also important to note that Graciosa is located on this route. Although *J. brevifolia* is extinct in Graciosa, this island may have constituted a stepping stone connecting the western group. The additional analysis of the *psb*A-*trn*H length polymorphism in 367 juniper trees also reflects a high dispersibility of *J. brevifolia* among the central islands and, to a lesser extent, some connectivity between the central and the eastern groups, which implies hopping a minimum distance of 139 km. In particular, the great connectivity among the central group may have had its peak during the colder phase of the last glaciation (∼18,000 years BP), when the sea level dropped more than 100 m [75], [76] and Pico and Faial formed a single landmass [77], [78].

The ease of colonization by the Azorean juniper is evidenced by its widespread range (all the islands except for Graciosa, where it is extinct), and has been reflected by the widespread distribution of ancestral haplotypes. This scenario correlates with recurrent inter-island colonization over long periods of time despite considerable geographic distances between island groups, and supports the hypothesis that Azorean endemic lineages have maintained substantial gene flow between islands [79]. However, this hypothesis has been recently challenged by results reported by Schaefer et al. [20], who analyzed the molecular variation of five endemic lineages (*Ammi*, *Euphorbia stygiana, Angelica lignescens, Azorina vidalii* and *Pericallis malviflora*) within the Azores range, and suggested that dispersal-mediated allopatry has been an extensive process in the archipelago, and considerable distances between some of the islands or island groups are effective barriers to gene flow. Overall, the total proportion of single-island haplotypes displayed by *J. brevifolia* (50%) was lower than that observed by Schaefer et al. [20] for the five endemic lineages studied (71%), which indicates higher connectivity of the Azorean junipers. In contrast, significant distribution of ancestral haplotypes of *J. brevifolia* parallels the widespread haplotype distribution of *Picconia azorica* across the islands [19], another wind pollinated and endozoochorous tree species endemic to the Azores [19], [80]. Using ISSRs and RAPDs, Silva et al. [81] also found that the largest portion of the *J. brevifolia* genetic variability resides within populations and among populations within islands, whereas the between island component is irrelevant. These results, together with the weak isolation-by-distance detected for *J. brevifolia* with the Mantel test, adds up to the idea of recurrent gene flow between island groups, supporting that the significant genetic differentiation observed is best interpreted by the temporal sequence of island emergence.

### Limited colonization of recent lineages

The widespread distribution of the ancestral haplotypes in the network may entail dispersal soon after *J. brevifolia* colonized the archipelago (Fig. 3), and furnishes molecular evidence for colonization related to island ages in the Azores. In contrast, six of the seven recent-most haplotypes are restricted to a single island, indicating that they may not have had enough time for inter-island dispersal. This pattern needs to be further investigated in the flora of the Azores given that a widespread distribution of ancient haplotypes, as opposed to a geographic restriction of the most recent (derived) ones, is also featured by *Picconia azorica* [19]. Alternatively, new colonization may have been prevented by the presence of already established junipers containing ancestral haplotypes. This finding is related to the ‘Darwin's naturalization hypothesis’, as recently tested by Schaefer et al. [9] for the Azorean flora. This hypothesis proposes that naturalization is more likely for aliens with no close relatives in the new land, due to lack of competitive exclusion [82] i. e. closely related species are more likely to have similar ecological niches due to common ancestry, and therefore would be competing for the same resources. Extending this hypothesis not only for closely related species but also for intraspecific lineages, our results suggest that colonization of *J. brevifolia* could have been hindered by the presence of early juniper lineages already occupying a similar ecological niche. Thus, restriction of six recent haplotypes to single islands may be due to the occurrence of habitat competition with early lineages or plant traits unfavorable for long-distance dispersal in relatively short periods of time.

### Traits promoting long-distance dispersal

The reproductive traits of *J. brevifolia* appear to have been favorable for long-distance dispersal. Feasibly, pollen flow among the present islands may have been relatively dynamic because of the prevailing winds. The Azores are usually under the influence of either tropical or polar maritime air masses, as a consequence of the seasonal drifting of the high-pressure Azores Anticyclone [83]. Furthermore, whenever the high-pressure center is dissipated or displaced, a polar atmospheric front shifts southwards, and several low-pressure fronts may sweep the whole archipelago. During the extended winter (October to March), the Azores region is frequently crossed by the North Atlantic storm-track [83]. As pollen shed occurs mainly during spring, the strong winds still frequent at this season could move pollen over long distances [84]. On the other hand, as *J. brevifolia* presents fleshy female cones edible for passerine birds, the gene flow estimated in this paper could be also due to the long-distance dispersal of seeds. A recent study on this juniper's seed dispersal system [31] revealed that birds, mainly blackbirds (*Turdus merula*) and blackcap warblers (*Sylvia atricapilla*), are active dispersal agents (frequency of occurrence of seeds in droppings: 81.1% and 6.1%, respectively). The question remains as to whether these birds are responsible for distribution of junipers in the Azores islands. Gut passage times are clearly different between them, *T. merula*'s being longer because of its larger size; consequently, this species defecates the seeds instead of regurgitating them, as it often happens in the smaller *S. atricapilla*. Since the emergence of the islands, successive and occasionally very explosive eruptions (e.g. in São Miguel, Terceira and Faial [85]) also may have promoted the movement of birds among islands in the attempt to escape from these disturbances. Furthermore, strong winds caused by the North Atlantic storm-track [83], may have promoted bird dispersal within the Azores archipelago. Therefore, blackbirds could have been largely responsible for the seed movements of at least the recent haplotypes.

### Concluding remarks

In summary, successful gene flow through pollen (anemophily) and seeds (ornithochory), may have promoted a relatively dynamic colonization by early junipers followed by a more parsimonious establishment of lineages. In addition, colonization success also depends on environmental suitability [2], [86], and the ability of the species to thrive in the habitat reached. In this respect, the Azores archipelago presents a remarkable habitat homogeneity and climatic stability –at least over the past 6,000 years [73] –, and *J. brevifolia* has been described as a pioneer species with a broad ecological range (0–1500 m.a.s.l), capable of colonizing recent substrates [21], [87]. In the long term, both ecological factors may have facilitated the establishment of new propagules, and thereby contributed to the successful colonization of *J. brevifolia* since island formation.

## Supporting Information

Table S1 **Taxon names, geographical area, locality, voucher information and GeneBank accession numbers for the** ***Juniperus*** **samples included in the phylogenetic analysis.** Coding numbers as in Fig. 2. Voucher abbreviations: BR: B. Rumeu collection numbers, as coded in the DNA Bank at the Jardín Botánico Canario ‘Viera y Clavijo’-Unidad Asociada CSIC; JM: J. Martínez voucher numbers; MA: herbarium of the Royal Botanic Garden of Madrid; E: Royal Botanic Garden, Edinburgh, Scotland, U.K. Taxonomy follows that of [6].
(PDF)

Table S2 **Plant material used for sequencing** ***pet*** **N-** ***psb*** **M,** ***trn*** **S-** ***trn*** **G and** ***trn*** **T-** ***trn*** **L from 72 samples.** N is the number of samples collected in each locality, voucher source indicates each tree sampled and H is the haplotype found. Voucher abbreviations: BR: B. Rumeu collection numbers, as coded in the DNA Bank at the Jardín Botánico Canario ‘Viera y Clavijo’- Unidad Asociada CSIC. Sequences of each haplotype were deposited in the GenBank.
(PDF)
