# Supplementary material for: The Colonization History of Juniperus brevifolia (Cupressaceae) in the Azores Islands
Source: PLoS One. 2011 Nov 16;6(11):e27697. doi: 10.1371/journal.pone.0027697 (PMC3218011; doi:10.1371/journal.pone.0027697)
Supplement: Table S1 — Taxon names, geographical area, locality, voucher information and GeneBank accession numbers for the Juniperus samples included in the phylogenetic analysis. Coding numbers as in Fig. 2. Voucher abbreviations: BR: B. Rumeu collection numbers, as coded in the DNA Bank at the Jardín Botánico Canario ‘Viera y Clavijo’-Unidad Asociada CSIC; JM: J. Martínez voucher numbers; MA: herbarium of the Royal Botanic Garden of Madrid; E: Royal Botanic Garden, Edinburgh, Scotland, U.K. Taxonomy follows that of [6]. (PDF) [file pone.0027697.s001.pdf]

Table S1. Taxon names, geographical area, locality, voucher information and GeneBank accession numbers for the *Juniperus* samples included in the phylogenetic analysis. Coding numbers as in Fig. 2. Voucher abbreviations: BR: B. Rumeu collection numbers, as coded in the DNA Bank at the Jardín Botánico Canario 'Viera y Clavijo'-Unidad Asociada CSIC; JM: J. Martínez voucher numbers; MA: herbarium of the Royal Botanic Garden of Madrid; E: Royal Botanic Garden, Edinburgh, Scotland, U.K. Taxonomy follows that of [6].

Coding no.	Taxon	Origin/Voucher	GenBank
1	*J. brevifolia*	Portugal, Azores, Santa Maria, Almagreira, BR4710	JF950948
2	*J. brevifolia*	Portugal, Azores, São Miguel, Serra da Tronqueira, BR4512	JF950949
3	*J. brevifolia*	Portugal, Azores, Terceira, Malha Grande, BR4546	JF950950
4	*J. brevifolia*	Portugal, Azores, São Jorge, R.F. Pico da Esperança, BR4450	JF950951
5	*J. brevifolia*	Portugal, Azores, Pico, Cachorro, BR4347	JF950952
6	*J. brevifolia*	Portugal, Azores, Pico, Lagoa do Capitão, Fiz, O. (Martínez and Vargas *in prep.*)	JF950953
7	*J. brevifolia*	Portugal, Azores, Faial, Caldeira, BR4415	JF950954
8	*J. brevifolia*	Portugal, Azores, Faial, Caldeira, Fiz, O. (Martínez and Vargas *in prep.*)	JF950955
9	*J. brevifolia*	Portugal, Azores, Flores, Alto da Cova, BR4663	JF950956
10	*J. brevifolia*	Portugal, Azores, Corvo, Lomba Redonda, BR4615	JF950957
11	*J. cedrus*	Spain, Canary Islands, Tenerife, La Orotava, Martínez, J. (Martínez and Vargas *in prep.*)	JF950958
12	*J. cedrus*	Spain, Canary Islands, La Palma, Pared de Roberto, BR5000	JF950959
13	*J. communis* var. *communis*	Spain, Burgos, Covarrubias, Vargas, P. (Martínez and Vargas *in prep.*)	JF950960
14	*J. communis* var. *communis*	Spain, Granada, Pico Trevenque, MA612184 (Martínez and Vargas *in prep.*)	JF950961
15	*J. communis* var. *communis*	France, WO-2111 (Mao et al. 2010)	HM024557
16	*J. communis* var. *depressa*	USA, New Mexico, BU-10936 (Mao et al. 2010)	HM024558
17	*J. communis* var. *saxatilis*	Spain, Madrid, La Pedriza, Martínez, J (Martínez and Vargas *in prep.*)	JF950962
18	*J. communis* var. *saxatilis*	Georgia, Caucasus, MA576696 (Martínez and Vargas *in prep.*)	JF950963
19	*J. communis* var. *saxatilis*	Pakistan, G.S.Miehe-3011 (Mao et al. 2010)	HM024559
20	*J. deltoides*	Turkey, WO-2002 (Mao et al. 2010)	HM024561
21	*J. formosana* var. *mairei*	China, Gansu, J.Q.Liu-1469 (Mao et al. 2010)	HM024568
22	*J. macrocarpa*	Spain, Cádiz, Barbate, Trafalgar Cape, Nieto, G.& Fuertes, J. (Martínez and Vargas in prep.)	JF950964
23	*J. macrocarpa*	Spain, Cádiz, Tarifa, JM5E8	JF950965
24	*J. macrocarpa*	Spain, Valencia, El Saler, Martínez, J. (Martínez and Vargas *in prep.*)	JF950966
25	*J. macrocarpa*	Spain, Cádiz, Chiclana, Eliçabe, L.M. (Martínez and Vargas *in prep.*)	JF950967
26	*J. macrocarpa*	Italy, Sardinia, Santa Teresa Gallura, Nieto, G.& Fuertes, J. (Martínez and Vargas *in prep.*)	JF950968
27	*J. macrocarpa*	Italy, Sicily, Ragusa, MA645785 (Martínez and Vargas *in prep.*)	JF950969
28	*J. maderensis*	Madeira, Fajã da Nogueira, BR6174	JF950970
29	*J. navicularis*	Portugal, Apostiça (Martínez and Vargas *in prep.*)	JF950971
30	*J. navicularis*	Portugal, Apostiça (Martínez and Vargas *in prep.*)	JF950972
31	*J. navicularis*	Portugal, Estremadura, MA565189 (Martínez and Vargas *in prep.*)	JF950973
32	*J. oxycedrus* var. *badia*	Spain, Huesca, Sierra de Guara, Vargas, P (Martínez and Vargas *in prep.*)	JF950974
33	*J. oxycedrus* var. *badia*	Spain, Madrid, Villalba, Martínez, J. (Martínez and Vargas *in prep.*)	JF950975
34	*J. oxycedrus* var. *badia*	Spain, Ciudad Real, Puebla de Don Rodrigo, MA615745 (Martínez and Vargas *in prep.*)	JF950976
35	*J. oxycedrus* var. *badia*	Spain, Jaén (Martínez and Vargas *in prep.*)	JF950977
36	*J. oxycedrus* var. *badia*	Spain, Jaén (Martínez and Vargas *in prep.*)	JF950978
37	*J. oxycedrus* var. *badia*	Morocco, Kjbel Kelti, Pérez, R. (Martínez and Vargas *in prep.*)	JF950979
38	*J. oxycedrus* var. *badia*	Morocco, Kjbel Kelti, Pérez, R. (Martínez and Vargas *in prep.*)	JF950980
39	*J. oxycedrus* var. *badia*	Turkey, Gümüshane, Torul, Herrero, A. et al. (Martínez and Vargas *in prep.*)	JF950981
40	*J. oxycedrus* var. *oxycedrus*	Spain, Granada, El Peñón, JM1E7	JF950982
41	*J. oxycedrus* var. *oxycedrus*	Spain, Granada, El Peñón, JM4E8	JF950983
42	*J. oxycedrus* var. *oxycedrus*	Spain, Balearic Islands, Menorca, Martínez, J. (Martínez and Vargas *in prep.*)	JF950984
43	*J. oxycedrus* var. *oxycedrus*	Greece, Lemo (Martínez and Vargas *in prep.*)	JF950985
44	*J. oxycedrus* var. *oxycedrus*	Greece, Lemo (Martínez and Vargas *in prep.*)	JF950986
45	*J. oxycedrus*	Turkey, Istanbul, Kartal, EOO138135 (Martínez and Vargas *in prep.*)	JF950987
46	*J. oxycedrus*	Greece, Kalavryta, Diakoftó, Vargas, P. (Martínez and Vargas *in prep.*)	JF950988
47	*J. oxycedrus*	Tunisia, Cap Bon, Sidi Daoud, Aldasoro et al. (Martínez and Vargas *in prep.*)	JF950989
48	*J. oxycedrus*	France, WO-2113 (Mao et al. 2010)	HM024581
49	*J. rigida* var. *conferta*	Cultivated (Spain, Pontevedra, Lourizán), MA547007 (Martínez and Vargas *in prep.*)	JF950990
50	*J. rigida* var. *conferta*	Japan, J.Q.Liu-TBG-JRIC (Mao et al. 2010)	HM024591
51	*J. rigida* var. *rigida*	Japan (Martínez and Vargas *in prep.*)	JF950991
52	*J. rigida* var. *rigida*	Japan, J.Q.Liu-TBG-JRIR (Mao et al. 2010)	HM024592
53	*J. taxifolia*	Japan, J.Q.Liu-TBG-JTA (Mao et al. 2010)	HM024602
54	*J. taxifolia*	Japan, Bonin Island (Martínez and Vargas *in prep.*)	JF950992
55	*J. taxifolia* var. *lutchuensis*	Japan (Martínez and Vargas *in prep.*)	JF950993
	OUTGROUP		
56	*J. drupacea*	Greece (Martínez and Vargas *in prep.*)	JF950994
57	*J. drupacea*	Greece (Martínez and Vargas *in prep.*)	JF950995
58	*J. drupacea*	Greece, BU-5651 (Mao et al. 2010)	HM024563
