# Supplementary material for: The Colonization History of Juniperus brevifolia (Cupressaceae) in the Azores Islands
Source: PLoS One. 2011 Nov 16;6(11):e27697. doi: 10.1371/journal.pone.0027697 (PMC3218011; doi:10.1371/journal.pone.0027697)
Supplement: Table S2 — Plant material used for sequencing pet N- psb M, trn S- trn G and trn T- trn L from 72 samples. N is the number of samples collected in each locality, voucher source indicates each tree sampled and H is the haplotype found. Voucher abbreviations: BR: B. Rumeu collection numbers, as coded in the DNA Bank at the Jardín Botánico Canario ‘Viera y Clavijo’- Unidad Asociada CSIC. Sequences of each haplotype were deposited in the GenBank. (PDF) [file pone.0027697.s002.pdf]

Table S2. Plant material of *J. brevifolia* used for sequencing *pet*N-*psb*M, *trn*S-*trn*G and *trn*T-*trn*L from 71 samples. Outgroup taxon (last row): *Juniperus oxycedrus* var. *oxycedrus*. N is the number of samples collected in each locality, voucher source indicates each tree sampled and H is the haplotype found. Voucher abbreviations: BR: B. Rumeu collection numbers, as coded in the DNA Bank at the Jardín Botánico Canario 'Viera y Clavijo'- Unidad Asociada CSIC. Sequences of each haplotype were deposited in the GenBank.

Geographical area/locality	N	Voucher-source	H	GenBank accession no. (petN-psbM / *trn*S-*trn*G / *trn*T-*trn*L)		
Portugal, Azores, Santa Maria, Almagreira	2	BR4710, BR4711	9	JF951004	JF951025	JF951039
Portugal, Azores, São Miguel, Monte Escuro	4	BR4496	8	JF951003	JF951020	JF951038
		BR4499, BR4506	11	JF951006	JF951026	JF951042
		BR4508	9	–	–	–
Portugal, Azores, São Miguel, Serra da Tronqueira	6	BR4512	12	JF951007	JF951027	JF951041
		BR4518, BR4541	9	–	–	–
		BR4523, BR4533	14	JF951009	JF951021	JF951043
		BR4543	10	JF951005	JF951029	JF951040
Portugal, Azores, Terceira, Malha Grande	3	BR4549	8	–	–	–
		BR4555	16	JF951011	JF951023	JF951045
		BR4563	13	JF951008	JF951028	JF951034
Portugal, Azores, Terceira, Pico Alto	3	BR4569	8	–	–	–
		BR4578	16	–	–	–
		BR4583	7	JF951002	JF951019	JF951033
Portugal, Azores, Terceira, Santa Bárbara	2	BR4590	17	JF951012	JF951024	JF951046
		BR4597	9	–	–	–
Portugal, Azores, Terceira, Fajãzinha	2	BR4605, BR4609	16	–	–	–
Portugal, Azores, São Jorge, Bocas do Fogo	2	BR4444	8	–	–	–
		BR4445	9	–	–	–
Portugal, Azores, São Jorge, R.F. Pico da Esperança	4	BR4450	14	–	–	–
		BR4455, BR4463	8	–	–	–
		BR4459	4	JF950999	JF951016	JF951036
Portugal, Azores, São Jorge, Serra do Topo	4	BR4468, BR4472, BR4490	8	–	–	–
		BR4474	15	JF951010	JF951022	JF951044
Portugal, Azores, Pico, Cachorro	1	BR4347	8	–	–	–
Portugal, Azores, Pico, Cerrado de Sonicas	1	BR4349	14	–	–	–
Portugal, Azores, Pico, Montanha	1	BR4358	4	–	–	–
Portugal, Azores, Pico, Baldios	1	BR4361	4	–	–	–
Portugal, Azores, Pico, Lagoa do Capitão	1	BR4368	8	–	–	–
Portugal, Azores, Pico, Curral Queimado-R.F. Prainha W	1	BR4373	8	–	–	–
Portugal, Azores, Pico, Curral Queimado-R.F. Prainha	1	BR4377	6	JF951001	JF951018	JF951032
Portugal, Azores, Pico, Piquete do Caveiro W	1	BR4386	4	–	–	–
Portugal, Azores, Pico, Manhenha	1	BR4392	8	–	–	–
Portugal, Azores, Faial, Quebrada	2	BR4394, BR4400	8	–	–	–
Portugal, Azores, Faial, Grotão	1	BR4405	8	–	–	–
Portugal, Azores, Faial, Caldeira	4	BR4407, BR4417	8	–	–	–
		BR4409	6	–	–	–
		BR4423	3	JF950998	JF951015	JF951035
Portugal, Azores, Faial, Cabeço dos Trinta	3	BR4425	4	–	–	–
		BR4438	8	–	–	–
		BR4442	5	–	–	–
Portugal, Azores, Flores, Lagoa	1	BR4660	8	–	–	–
Portugal, Azores, Flores, Alto da Cova	2	BR4663, BR4675	8	–	–	–
Portugal, Azores, Flores, Pico da Casinha	1	BR4680	8	–	–	–
Portugal, Azores, Flores, Caldeirães	1	BR4685	8	–	–	–
Portugal, Azores, Flores, Morro Alto e Pico da Sé	2	BR4689	5	JF951000	JF951017	JF951037
		BR4691	14	–	–	–
Portugal, Azores, Flores, Caldeira Funda e Rasa	2	BR4696	2	JF950997	JF951014	JF951031
		BR4700	8	–	–	–
Portugal, Azores, Flores, Fajãzinha	1	BR4706	8	–	–	–
Portugal, Azores, Corvo, Lomba Redonda	6	BR4610, BR4628	14	–	–	–
		BR4615, BR4633, BR4639	8	–	–	–
		BR4644	6	–	–	–
Portugal, Azores, Corvo, Cabeçeira	2	BR4647	14	–	–	–
		BR4652	16	–	–	–
Portugal, Azores, Corvo, Alqueve	2	BR4655, BR4658	14	–	–	–
OUTGROUP						
Greece, Lemo	1	Martínez and Vargas in prep.	1	JF950996	JF951013	JF951030
